# Poster Session II - A232 INFLAMMATORY BOWEL DISEASE IS ASSOCIATED WITH AN INCREASED RISK OF END-STAGE RENAL DISEASE: A POPULATION-BASED COHORT STUDY

**DOI:** 10.1093/jcag/gwaf042.232

**Published:** 2026-02-13

**Authors:** O Sattayalertyanyong, Z Nugent, B Barr, K Bernstein, C N Bernstein

**Affiliations:** Mahidol University Faculty of Medicine Siriraj Hospital, Bangkok, Bangkok, Thailand; University of Manitoba Max Rady College of Medicine, Winnipeg, MB, Canada; University of Manitoba Max Rady College of Medicine, Winnipeg, MB, Canada; University of Manitoba Max Rady College of Medicine, Winnipeg, MB, Canada; University of Manitoba Max Rady College of Medicine, Winnipeg, MB, Canada

## Abstract

**Background:**

Inflammatory bowel disease (IBD) patients frequently develop extraintestinal manifestations, including renal complications. However, the incidence and risk factors for end-stage renal disease (ESRD) in IBD remain poorly characterized.

**Aims:**

This study aims to assess the incidence of ESRD in IBD, compare its risk with the general population, and identify predictors of ESRD.

**Methods:**

This was a retrospective population-based cohort study using the University of Manitoba IBD Epidemiology Database linked to administrative healthcare data from 1984-2023 (12,639 IBD patients matched with 126,180 controls). ESRD was defined as requiring any two claims for outpatient dialysis within a 1-year period. Predictive factors were identified using proportional hazard regression and nested case-control logistic regression analyses.

A232 Table 1: Baseline characteristic of all participants in cohort

**Results:**

IBD patients had significantly higher incidence of dialysis (1.48% vs 0.82%, p < 0.0001) and kidney transplantation (0.25% vs 0.10%, p < 0.0001) than controls. IBD was an independent predictor of ESRD (HR = 1.53, 95% CI 1.23-1.90), with stronger associations in CD (HR = 1.93, 95% CI 1.39-2.68) than UC (HR = 1.28, 95% CI 0.96-1.72). Traditional risk factors including diabetes (HR = 3.82, 95% CI 3.22-4.53), hypertension (HR = 1.91, 95% CI 1.58-2.30), and congestive heart failure (HR = 6.37, 95% CI 5.25-7.72) remained strongly predictive. Within the IBD population, oral steroid treatment (OR = 2.0, 95%CI 1.06-3.81), allopurinol use (OR = 4.06, 95% CI 1.49-11.1), and bowel surgery (OR = 2.77, 95% CI 1.51-5.09) significantly predicted dialysis initiation

**Conclusions:**

IBD increases ESRD risk by nearly 50%, with CD showing greater risk than UC. Bowel surgery, allopurinol use, and oral steroid therapy are important predictors, emphasizing the necessity of careful renal monitoring in IBD patients.

Kaplan–Meier survival curves showing the cumulative incidence of all-cause ­mortality during follow-up among patients with Crohn’s disease (CD), ulcerative colitis (UC), and controls after initiation of dialysis.

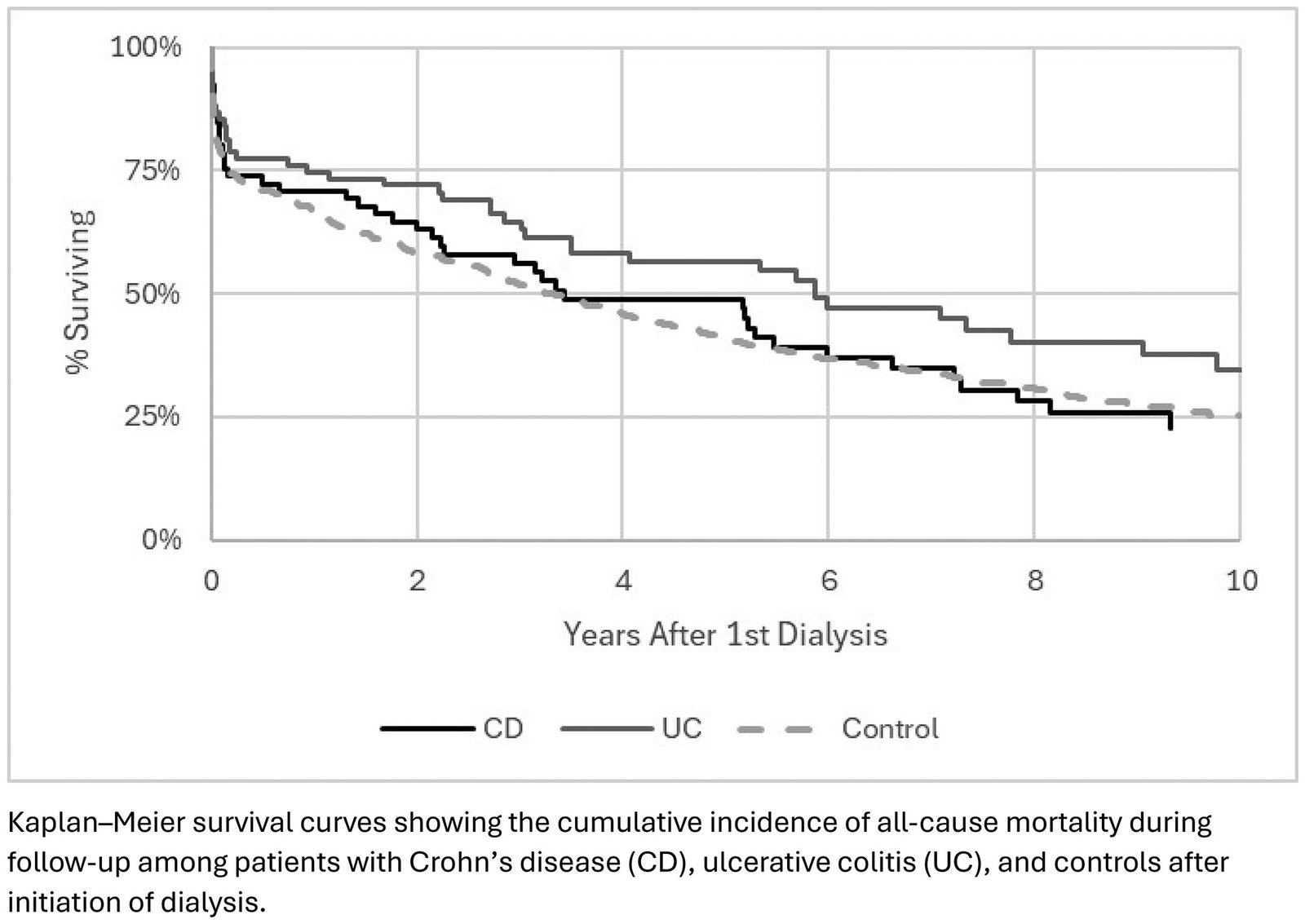

None

**Table:** 

Variables	IBD case (n = 12639)	control (n = 126180)	P-value	CD case (n = 5167)	control (n = 51576)	P-value	UC case (n = 7472)	control (n = 74064)	P-value
Male gender(%)	46.9			44.5			48.5		
Age at IBD diagnosis(IQ)	38(26-55)			34(23-50)			41(28-57)		
Hypertension	19.8	10.1	0.049	15.7	15.5	0.60	22.6	21.6	0.041
Diabetes	6.4	6.5	0.76	4.8	5.3	0.15	7.6	7.4	0.53
Hyperlipidemia	18.3	18	0.47	12.6	14.2	0.0016	22.2	20.7	0.0021
Renal stone	3.8	2.5	<0.0001	3.4	2.1	<0.0001	4.1	2.8	<0.0001
Congestive heart failure	3.2	2.4	<0.0001	2.2	1.7	<0.0001	3.9	2.9	<0.0001
Myocardial infarction	2.5	2	<0.0001	1.8	1.4	0.0248	3	2.3	0.0005
Peripheral Vascular	2.6	1.4	<0.0001	1.5	1	0.0013	3.4	1.6	<0.0001
Cerebrovascular	5	4.3	0.0002	3.7	3.3	0.12	5.9	4.9	0.0005
Connective tissue	1.8	1	<0.0001	1.4	0.6	<0.0001	2.2	1.2	<0.0001
Renal disease	2.3	1.3	<0.0001	1.7	0.9	<0.0001	2.8	1.6	<0.0001
Malignancy	9.3	6.8	<0.0001	8	5.3	<0.0001	10.2	7.9	<0.0001
Charlson Comorbidity Index = 0 (%)	83.6	89.7	<0.0001	85.1	91.8	<0.0001	82.5	88.2	<0.0001
History of Dialysis(%)	1.48	0.82	<0.0001	1.49	0.79	<0.0001	1.47	0.84	<0.0001
Median age of dialysis(IQ)	60(47-71)	61(49-71)	0.7	62(47-74)	58(45-68)	0.055	59(48-70)	63(52-772)	0.033
Kidney transplant(%)	0.25	0.1	<0.0001	0.17	0.1	0.11	0.29	0.1	<0.0001

